# Diagnosis and surgical management of an acquired cervical tracheoesophageal fistula in a Shih Tzu following foreign body removal

**DOI:** 10.1186/s13028-025-00842-5

**Published:** 2025-12-13

**Authors:** Hannelore De porte, Bart Van Goethem

**Affiliations:** 1 AniCura Haaglanden Rijswijk, Frijdastraat 20a, Rijswijk, NL-2288 EZ The Netherlands; 2 Small Animal Teaching Hospital, Faculty of Veterinary Medicine, Ghent University, Salisburylaan 133, Merelbeke, B-9820 Belgium

**Keywords:** Case report, Endoscopy, Esophageal foreign body, Tracheoesophageal fistula

## Abstract

**Background:**

Esophagorespiratory fistulae are rare pathological communications between the esophagus and the respiratory tract. While acquired bronchoesophageal fistulae have been sporadically documented in dogs, tracheoesophageal fistulae, particularly in the cervical region, are exceptionally rare. This case report is the first to describe an acquired cervical tracheoesophageal fistula in a dog following esophageal foreign body retention. The case highlights the clinical course, diagnostic challenges, and successful surgical management, expanding the current understanding of this uncommon condition in veterinary medicine.

**Case presentation:**

A 6.5-year-old male Shih Tzu was referred for persistent hyporexia, vomiting, and moist cough following endoscopic removal of a bone lodged at the thoracic inlet. Despite medical management for esophagitis and aspiration pneumonia, the dog exhibited progressive respiratory signs and recurrent pneumonia. An esophagorespiratory fistula was suspected based on the ability to provoke coughing by liquid ingestion and tracheal palpation. Endoscopic examination confirmed a defect in the esophageal wall with direct communication to the tracheal lumen, consistent with a cervical tracheoesophageal fistula. Surgical repair was performed via a cervical ventral midline approach, involving separate closure of the tracheal and esophageal defects and reinforcement using a bipedicle sternohyoid muscle flap. Recovery was uneventful, and the dog remains asymptomatic at 12-month follow-up.

**Conclusions:**

This case underscores the importance of considering a tracheoesophageal fistula in dogs presenting with persistent or recurrent gastrorespiratory signs following esophageal foreign body removal. Diagnosis can be challenging due to the rarity of the condition and its non-specific clinical presentation. Endoscopy proved to be a safe and effective diagnostic tool and avoided the risk of aspiration associated with contrast studies. The surgical technique, including muscle flap interposition, mirrors approaches used in human medicine, where similar strategies are applied to prevent recurrence. Comparative insights with human cases highlight the translational value of interspecies knowledge, especially in understanding pathogenesis, refining diagnostics, and optimizing treatment. Continued reporting and awareness of tracheoesophageal fistula in veterinary patients are essential to improve outcomes and explore minimally invasive alternatives.

## Background

Esophagorespiratory fistulae (ERFs) are rare anatomical disorders characterized by a patent connection between the esophagus and the respiratory tract. They can be either congenital or acquired, and are classified as tracheoesophageal (TEF), bronchoesophageal (BEF), and esophagopulmonary fistulae based on their anatomic location [1–3]. In dogs, ERFs are typically caused by the retention of an ingested foreign body (FB), usually a bone, that causes progressive focal esophageal necrosis and thereby fistulation to the adjacent part of the respiratory tract. Other etiologies include trauma (e.g. bite wounds), (periesophageal) inflammation, focal infection, neoplasia, or iatrogenic trauma [4–7]. As most esophageal FB obstructions occur caudal to the heart, BEFs are the predominant type of ERF diagnosed in dogs [1, 5]. Whereas acquired BEF are relatively well-documented, this case report describes an acquired cervical TEF, a rare variant not previously documented in canine patients (Table 1). By detailing its clinical course, diagnostic challenges, and management, this report broadens current understanding of TEFs in dogs and highlights key considerations for early recognition and intervention.

**Table 1:** Available veterinary literature on tracheoesophageal fistula in dogs

Author(s)	Case Description	Fistula Characteristics	Treatment	Outcome
Shebitz (1960)*	Radiographic appearance of a TEF	Acquired (FB)	/	/
Stogdale et al. (1977)	2-year-old Yorkshire Terrier with chronic history of coughing, dysphagia, and vomiting	Congenital; thoracic (dorsal to heart, just caudal to aperture of cranial bronchus); 3 mm diameter	Right-sided thoracotomy (6th IC space); fistula ligation	Dog died on recovery
Dodman & Baker (1978)	1.5-year-old Yorkshire Terrier with chronic history of retching, coughing, and anorexia after ingestion of chop bones	Acquired (FB); thoracic (cranial to heart base); 3 mm diameter	Right-sided thoracotomy (4th IC space); fistula resection and closure of defects with single interrupted sutures	Resolution of clinical signs 2 months post-op with some tracheal narrowing at the former fistula site and localized opacity of the lung field cranial to the heart
Kaminen et al. (2014)	7-month-old Spanish Water Dog with recurrent respiratory symptoms	Congenital; thoracic (tracheal bifurcation); 0.5–3 mm diameter	Right-sided thoracotomy (6th IC space); fistula ligation and transection	No recurrence of clinical signs at 10 months post-op
Bottero et al. (2019)	8-month-old Maremma Sheepdog with chronic cough	Congenital; thoracic (tracheal bifurcation); 0.4 mm diameter	Endoscopic laser ablation	Fistula closure confirmed by endoscopy at 1 month post-op and dog clinically normal at 6 months post-op

Original publication not digitally accessible (*Tierärztliche Umschau*,* 1960*)

Case details are based on secondary references (Baker, 1966; Thrall, 1973; Keane et al., 1983; Kaminen et al., 2014) and should be interpreted with this limitation in mind

## Case presentation

A 6.5-year-old intact male Shih Tzu was referred for evaluation of chronic gastrointestinal and respiratory problems. Three weeks prior to referral, a bone that was entrapped in the esophagus at the level of the thoracic inlet was removed via endoscopy-assisted retrieval. Despite the initiation of medical treatment for esophagitis, the dog developed persistent hyporexia and vomiting, a moist non-productive cough, and occasional respiratory distress. Complete blood count (CBC) indicated severe neutrophilic leukocytosis, and thoracic radiographs confirmed the presence of focal aspiration pneumonia (AP). Antibiotics and anti-emetics were added to the treatment.

Follow-up thoracic radiography one week later showed cessation of the pneumonia. The owner reported marked improvement in the dog’s condition, with coughing occurring only after ingesting liquids. Antibiotic treatment was continued for an additional two weeks, consistent with current guidelines for managing AP in dogs [8].

Six weeks after removal of the esophageal FB, the dog presented alert but cachectic. Spontaneous coughing with mucoid discharge from the nose and mouth was evident. Noteworthy, coughing could be provoked upon tracheal palpation and exacerbated following the ingestion of liquids. Vital signs were within normal limits, however, CBC revealed mild leukocytosis (16.49 × 10⁹/L; RR: 5.05–16.76 × 10⁹/L) with a regenerative left shift and monocytosis (2.48 × 10⁹/L; RR: 0.16–1.12 × 10⁹/L), indicative of an active inflammatory response. Serum lactate was moderately elevated (5.66 mmol/L; RR: 0.5–2.5 mmol/L), and C-reactive protein was markedly increased (58 mg/L; RR: 0–10 mg/L), confirming the presence of systemic inflammation. Thoracic radiographs again showed signs of AP. Given the ongoing gastrorespiratory symptoms, differential diagnoses included megaesophagus, gastroesophageal reflux, dysphagia due to pharyngeal or laryngeal inflammation, cricopharyngeal achalasia, esophageal stricture or tumor, and ERF. Diagnostic endoscopy of the esophagus and trachea was selected for further evaluation.

The dog was premedicated intravenously (IV) with 2 µg/kg medetomidine (Domitor, 1 mg/mL, Orion Pharma, Espoo, Finland) and 0.15 mg/kg butorphanol (Dolorex, 10 mg/mL, Intervet International BV, Boxmeer, The Netherlands), and anesthesia was maintained with boli of 2 mg/kg alfaxalone (Alfaxan, 10 mg/mL, Zoetis Belgium, Louvain-La-Neuve, Belgium) to effect. A 5.5-mm endotracheal (ET) tube was used to ensure a patent airway during the procedure. Examination of the mouth and laryngeal region showed no gross abnormalities.

**Fig. 1:**
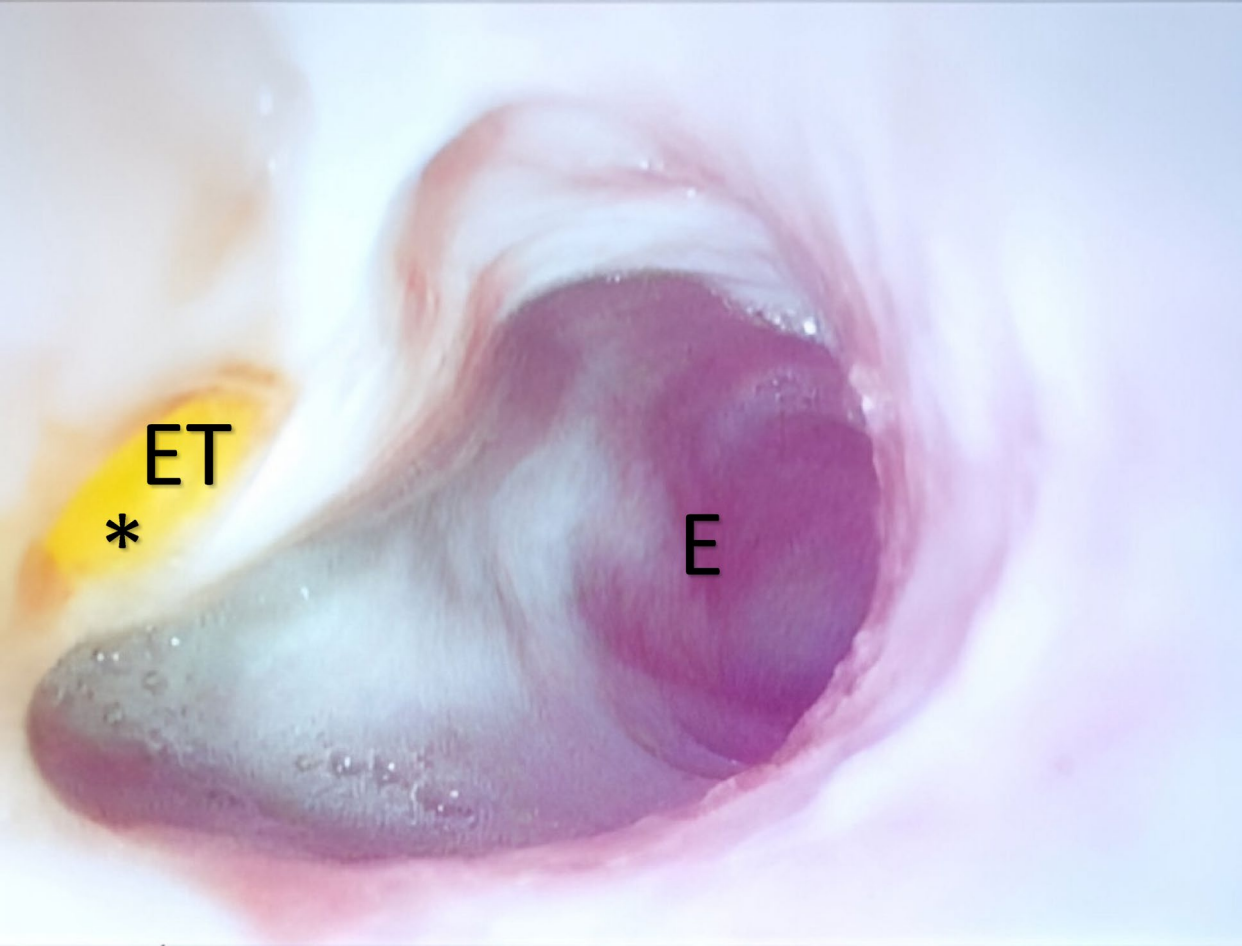
Endoscopic findings. Endoscopic image of the cervical esophagus (E) demonstrating a well-demarcated defect (*) in the esophageal wall, consistent with a tracheoesophageal fistula. Visualization of the colored endotracheal tube (ET) through the defect confirms a pathological communication between the esophageal and tracheal lumina

Diagnostic endoscopy with an 8.9-mm flexible gastroendoscope (Dr Fritz), revealed a defect in the esophagus at the level of the thoracic inlet, through which the endotracheal tube could be visualized (Fig. 1). No further abnormalities were detected in the esophagus or stomach. A diagnosis of a TEF was made, and surgical correction was scheduled. In the meantime, antibiotic treatment was continued, and the owners were instructed on feeding practices to minimize the risk of AP.

On the day of surgery, the dog received intramuscular premedication with 2 µg/kg medetomidine and 0.2 mg/kg methadone (Comfortan, 10 mg/mL, Dechra Regulatory BV, Bladel, The Netherlands), followed by IV induction with 0.2 mg/kg diazepam (Ziapam, 5 mg/mL, Domes Pharma, Pont-du-Chateau, France) and 5 mg/kg ketamine (Nimatek, 100 mg/mL, Dechra Regulatory BV, Bladel, The Netherlands). At induction, 2 mg/kg robenacoxib (Onsior, 20 mg/mL, Elanco Europe Ltd., Basingstoke, UK) and 12.5 mg/kg amoxicillin-clavulanic acid (Noroclav, Norbook Laboratories (Ireland) Limited, Monaghan, Ireland) were administered subcutaneously. General anesthesia was maintained with 2% isoflurane (Iso-Vet, 1000 mg/g, Piramal Critical Care BV, Voorschoten, The Netherlands) in oxygen and vital signs were monitored with pulse oximetry and electrocardiography.

**Fig. 2:**
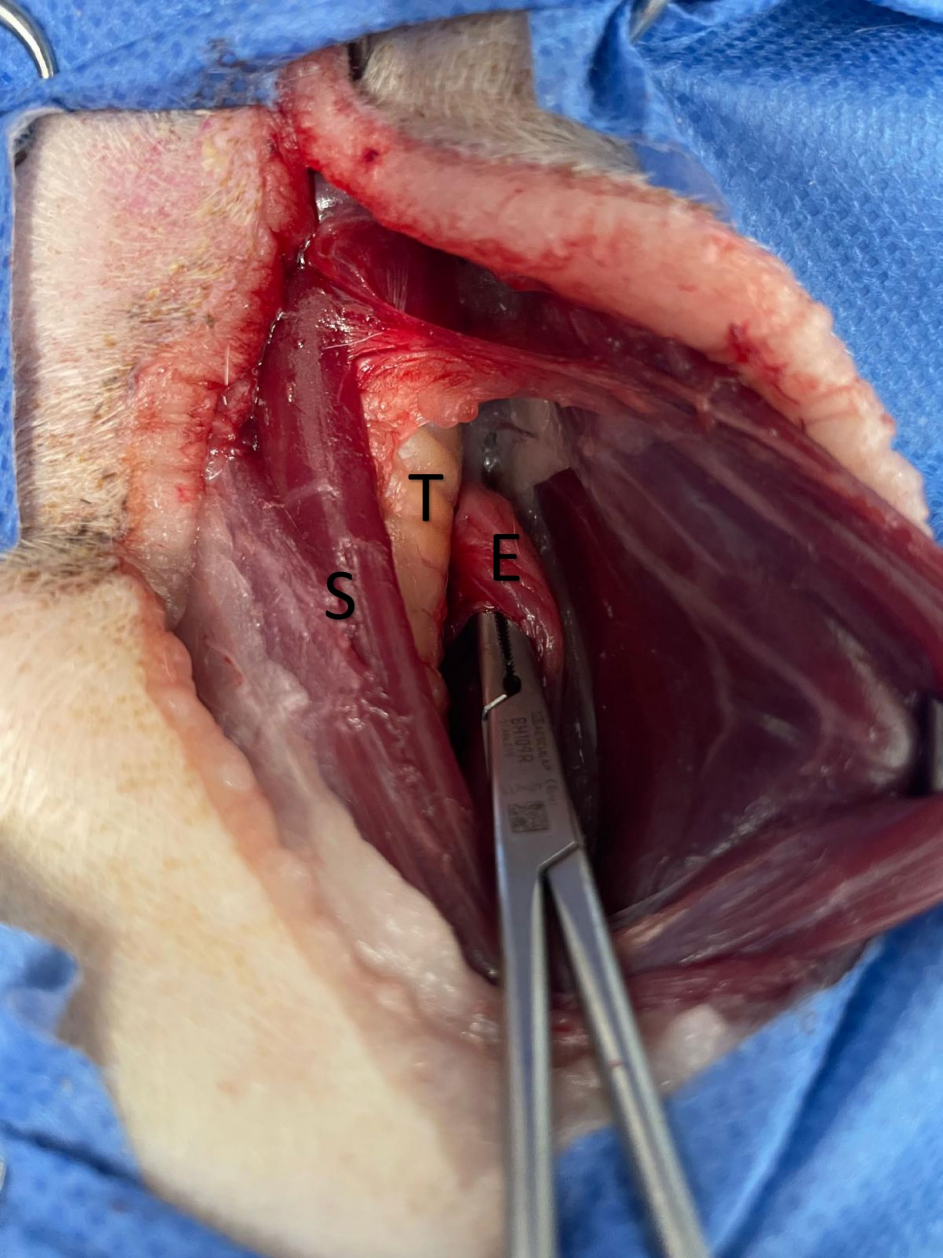
Surgical findings. Intraoperative view obtained via a cervical ventral midline approach, demonstrating separation of the esophagus (E) from the trachea (T) following dissection. The esophageal defect, corresponding to the site of tracheoesophageal fistula transection, is located on the right lateral wall and delineated by the insertion of a needle holder to indicate its position and extent. The sternohyoid muscle (S) is also visible in situ prior to its elevation as a vascularized bipedicle flap for interposition between the trachea and esophagus

A ventral midline approach to the cervical trachea was performed as previously described [9]. A defect consistent with a TEF was identified between the dorsal tracheal muscle and the right lateral esophageal wall. The trachea and esophagus were separated using a combination of sharp and blunt dissection, with careful preservation of the recurrent laryngeal nerves and tracheal vascular supply (Fig. 2). The resulting defects were debrided to ensure healthy, viable margins. The tracheal defect was closed longitudinally using interrupted horizontal mattress sutures with 4 − 0 polydioxanone (PDS II, Ethicon), providing tension relief and robust wound edge apposition. In contrast, the esophageal defect was closed transversely to optimize luminal patency, using a single-layer, simple continuous appositional suture pattern with the same suture material. To reinforce the repair and provide physical separation between both suture lines, a vascularized bipedicle sternohyoid muscle flap was elevated and interposed between the trachea and esophagus. The flap was sutured to the dorsolateral aspect of the trachea using simple interrupted 4 − 0 polydioxanone sutures. Routine closure of the cervical incision was performed in four anatomical layers.

Postoperative recovery was uneventful, and the patient was discharged the following day with a six-day oral course of 2 mg/kg robenacoxib (Onsior, Elanco Europe Ltd., Basingstoke, UK) SID and a two-week oral course of 12.5 mg/kg amoxicillin-clavulanic acid (Clavaseptin, Vetoquinol NV/SA, Niel, Belgium) BID.

At the two-week postoperative check-up, the dog exhibited increased energy levels, weight gain, and cessation of any respiratory or feeding difficulties. Tracheal palpation no longer elicited coughing or nasal discharge. A follow-up telephone check-up at 12 months postoperatively revealed no recurrence of clinical signs.

### Discussion and conclusions

The dog described in this case report developed a cervical TEF, a rare pathological connection between the trachea and the esophagus, following retention of a FB (bone or bone material) in the esophagus. Although clinical signs consistent with TEF emerged shortly after FB removal, diagnosis of this complication was delayed by six weeks.

In veterinary medicine, diagnosing TEFs remains challenging due to the rarity of the condition, its variable and nonspecific clinical presentation, and the lack of standardized diagnostic protocols [10–13]. Clinical signs vary depending on the location, size, and rate of the fistula formation. Moreover, symptoms like chronic cough, recurrent pneumonia, dysphagia, and weight loss are often mistakenly attributed to more common respiratory or gastrointestinal disorders, delaying accurate diagnosis [1, 14]. Early recognition is crucial to prevent further tracheal injury and secondary pulmonary complications resulting from chronic aspiration [15, 16].

In dogs, acquired TEFs most commonly develop following prolonged retention of a bony FB within the esophagus [6, 10, 11]. The sustained presence of the foreign material induces localized pressure necrosis that can progress to full-thickness esophageal and tracheal perforation. When fast-occurring, this typically results in esophageal perforation into the periesophageal structures. However, in case of a gradual, slow-occurring necrosis, continued inflammation and remodeling of the surrounding tissues will result in fistula formation [1, 17]. Experimental validation of this pathophysiology was demonstrated by *Gao et al.* [18]. , who successfully induced TEFs in dogs via transesophageal and transtracheal placement of magnets, generating localized pressure necrosis and subsequent fistula formation within 4–6 days.

In human medicine, diagnostic workup for TEF typically involves a combination of radiographic, fluoroscopic, and endoscopic techniques. Although there are no formal guidelines, most experts agree that esophagography and endoscopy are essential for establishing the diagnosis and facilitating preoperative planning [12, 13]. In human patients, contrast-enhanced esophagography has been reported to identify fistulae in roughly 70–80% of cases, but may fail due to variable patency, small size, or oblique course of the fistula [19, 20]. A critical drawback of contrast studies - both in human and veterinary patients - is the potential risk associated with barium sulphate aspiration, which can lead to pulmonary granulomas and chronic respiratory complications [21–23]. Non-ionic iodinated contrast agents such as iopydol offer a safer alternative but may have reduced sensitivity due to lower radiopacity. In veterinary patients, their use is further complicated because of poor voluntary ingestion [24–27].

Endoscopic evaluation, including esophago- and tracheobronchoscopy, is a valuable diagnostic tool in both human and veterinary medicine, as it provides direct visualization of the fistula, allowing for precise localization and characterization of the defect. However, the presence of mucosal oedema, esophageal debris, and fistula occlusion by inflammatory tissue can reduce diagnostic accuracy [12]. Adjunctive use of orally administered methylene blue has been described in human medicine to enhance fistula detection [28–30], although its utility remains anecdotal and has not been widely adopted in veterinary practice.

The use of computed tomography (CT) has gained increasing interest over the years, offering detailed anatomical visualization and aiding in cases where contrast esophagography is inconclusive or contraindicated. Despite its potential, the sensitivity of CT in diagnosing TEFs remains a topic of debate, and its routine use in veterinary medicine might still be limited due to cost [12, 14, 20].

Recent studies have explored alternative diagnostic methods, such as capnography with CO_2_ insufflation, which may improve TEF detection when combined with traditional modalities [31]. While this approach has shown potential in human medicine, its clinical utility and sensitivity require further validation before broader adoption.

In the present case, esophagoscopy was selected as the diagnostic method based on strong clinical suspicion and practical considerations, including financial constraints. This approach allowed for direct visualization of the esophageal defect while avoiding the risks associated with contrast aspiration.

In human medicine, surgical closure is still considered the treatment of choice for non-malignant TEFs, especially those located in the cervical area [12, 16, 32]. Although surgery offers the potential for curative treatment, it carries notable morbidity, including the risk of recurrent laryngeal nerve injury and fistula recurrence [16, 33, 34]. Consequently, there has been increasing interest in less invasive alternatives, such as endoscopic stenting, in order to reduce the complications associated with open surgery. Endoluminal stents, however, have a high tendency to migrate and might even cause an increase in the size of the fistula [2, 16], making them less favorable as a first treatment option in case of a resectable fistula. Additional non-surgical options described in human literature [12, 35] include the use of tissue adhesives (e.g. fibrin glue, cyanoacrylate), endoscopic clips (over-the-scope clipping) [36], vascular closure devices such as the Amplatzer Occluder [37], endoluminal vacuum-assisted closure therapy [3], and laser treatment [11]. However, these approaches have limited supporting evidence, primarily derived from case reports, and are yet to be extensively researched.

Veterinary management of ERFs has traditionally mirrored human practices, but requires adaptation due to anatomical and procedural differences. In the case presented, open surgical repair was chosen based on the accessible cervical location and the relatively large size of the fistula, making it less suitable for alternative treatments such as fibrin glue (< 5 mm), laser (< 6 mm) [11], and over-the-scope clipping (< 10 mm) [36]. A bipedicle muscle flap (left sternohyoid muscle flap) was used to support primary closure of the fistula defect. The additional blood supply and addition of a tissue layer between the suture lines may have helped prevent fistula recurrence in case of leakage and promotes healing by improving local circulation [38, 39].

This case of an acquired cervical TEF in a dog highlights the challenges and considerations in diagnosing and treating this rare condition. Endoscopy could successfully diagnose the condition and surgical resection of the fistula provided curative treatment. This case further underscores the need to actively consider a TEF in case of a retained foreign body and subsequent gastrorespiratory complaints. Further research and case documentation are needed to refine diagnostic protocols and treatment options for this rare but clinically relevant condition.

## Data Availability

Not applicable.

## Abbreviations

AP: aspiration pneumonia
BEF: bronchoesophageal fistula
CBC: complete blood count
CT: computed tomography
ERF: esophagorespiratory fistula
FB: foreign body
IC: intercostal
IV: intravenous
TEF: tracheoesophageal fistula

## References

[1] Della Ripa MA, Gaschen F, Gaschen L, Cho DY. Canine bronchoesophageal fistulas: case report and literature review. Compend Contin Educ Vet. 2010;32:E1. 20949418

[2] Bixby BA, Maddock SD, Reddy CB, Ansari SA. Acquired esophago-respiratory fistulae in adults. Shang Chest. 2020;4:4. 10.21037/shc.2019.11.06.

[3] Kamaleddine I, Popova M, Alwali A, Schafmayer C. Endoscopic vacuum therapy for treating an esophago-pulmonary fistula after esophagectomy: A case report and review of the literature. Visc Med. 2023;39:18–24. 10.1159/000529725. 37125383 10.1159/000529725 PMC10130739

[4] Hedlund CS. Surgery of the esophagus. In: Fossum TW, editor. Small Animal Surgery. 2nd edition. Missouri, USA: Mosby; 2002. pp. 307–37.

[5] Nawrocki MA, Mackin AJ, McLaughlin R, Cantwell HD. Fluoroscopic and endoscopic localization of an esophagobronchial fistula in a dog. J Am Anim Hosp Assoc. 2003;39:257–61. 10.5326/0390257. 12755199 10.5326/0390257

[6] Jergens AE. Diseases of the esophagus. In: Ettinger SJ, Feldman EC, editors. Textbook of Veterinary Internal Medicine. 6th edition. Philadelphia, USA: Elsevier Saunders; 2015. pp. 1298–1310.

[7] Johnson LR. Diseases of the small airways. In: Ettinger SJ, Feldman EC, editors. Textbook of Veterinary Internal Medicine. 6th edition. Philadelphia, USA: Elsevier Saunders; 2015. pp. 1233–9.

[8] Fernandes Rodrigues N, Giraud L, Bolen G, Fastrès A, Clercx C, Gommeren K, et al. Antimicrobial discontinuation in dogs with acute aspiration pneumonia based on clinical improvement and normalization of C-reactive protein concentration. JVIM. 2022;36:1082–8. 10.1111/jvim.16405. 10.1111/jvim.16405 PMC9151469 35348224

[9] Hayes AM (Durant), Seibert R, Sura PA. Trachea and bronchi. In:, Johnston SA, Tobias KM, editors. Veterinary Surgery: Small Animal Expert Consult. 2nd edition. Philadelphia, USA: Elsevier Saunders; 2017. pp. 1963–83.

[10] Dodman NH, Baker GJ. Tracheo-oesophageal fistula as a complication of an esophageal foreign body in the dog: A case report. JSAP. 1978;19:291–6. 10.1111/j.1748-5827.1978.tb05494.x. 10.1111/j.1748-5827.1978.tb05494.x 661240

[11] Bottero E, Manassero E, De Lorenzi D. Diode laser treatment in a case of congenital tracheoesophageal fistula in a young dog. Can Vet J. 2019;60:472–6. 31080257 PMC6463437

[12] Kim HS, Khemasuwan D, Diaz-Mendoza J, Mehta AC. Management of tracheo‐oesophageal fistula in adults. ERR. 2020;29:200094. 10.1183/16000617.0094-2020. 10.1183/16000617.0094-2020 PMC9488631 33153989

[13] Ciecierega T, Sharaiha R. Endoscopic treatment of tracheoesophageal fistula: use of stents and endoclips. Curr Chall Thorac Surg. 2022;4:24. 10.21037/ccts-20-155.

[14] Kaminen PS, Viitanen SJ, Lappalainen AK, Kipar A, Rajamäki MM, Laitinen-Vapaavuori OM. Management of a congenital tracheoesophageal fistula in a young Spanish water dog. BMC Vet Res. 2014;10:16. 10.1186/1746-6148-10-16. 24423070 10.1186/1746-6148-10-16 PMC3895692

[15] Burt M, Diehl W, Martini N, Bains MS, Ginsberg RJ, McCormack PM, et al. Malignant esophagorespiratory fistula: management options and survival. Ann Thorac Surg. 1991;52:1222–9. 10.1016/0003-4975(91)90005-b. 1755674 10.1016/0003-4975(91)90005-b

[16] Hasan L, Sharma B, Goldenberg SA. Acquired tracheoesophageal fistulas: A case report and review of diagnostic and management challenges. Cureus. 2022;14:e23324. 10.7759/cureus.23324. 35464543 10.7759/cureus.23324 PMC9015068

[17] Salisbury SK, Forbes S, Blevins WE. Peritracheal abscess associated with tracheal collapse and bilateral laryngeal paralysis in a dog. J Am Vet Med Assoc. 1990;196:1273–5. 2332374

[18] Gao Y, Wu RQ, Lv Y, Yan XP. Novel magnetic compression technique for establishment of a canine model of tracheoesophageal fistula. World J Gastroenterol. 2019;25:4213–21. 10.3748/wjg.v25.i30.4213. 31435174 10.3748/wjg.v25.i30.4213 PMC6700694

[19] Couraud L, Ballester MJ, Delaisement C. Acquired tracheoesophageal fistula and its management. Semin Thorac Cardiovasc Surg. 1996;8:392–9. 8899926

[20] Ayaz E, Haliloglu M. Radiologic diagnosis of tracheoesophageal fistula in children. Curr Chall Thorac Surg. 2022;4:25. 10.21037/ccts-20-161.

[21] Ueha R, Nativ-Zeltzer N, Sato T, Goto T, Nito, Tsunoda K, et al. Chronic inflammatory response in the rat lung to commonly used contrast agents for videofluoroscopy. Laryngoscope Investig Otolaryngol. 2019;4:335–40. 10.1002/lio2.269. 31236468 10.1002/lio2.269 PMC6580069

[22] Ueha R, Nativ-Zeltzer N, Sato T, Goto T, Yamauchi A, Belafsky PC, et al. The effects of barium concentration levels on the pulmonary inflammatory response in a rat model of aspiration. Eur Arch Otorhinolaryngol. 2019;277:189–96. 10.1007/s00405-019-05666-4. 31555920 10.1007/s00405-019-05666-4

[23] Yan GW, Deng JF, Bhetuwal A, Yang GQ, Fu QS, Chen H, et al. A case report and literature review of barium sulphate aspiration during upper Gastrointestinal examination. Medicine. 2017;96:e8821. 10.1097/MD.0000000000008821. 29381987 10.1097/MD.0000000000008821 PMC5708986

[24] Herrtage ME, Dennis R. Contrast media and their use in small animal radiology. JSAP. 1987;28:1105–14. 10.1111/j.1748-5827.1987.tb01335.x.

[25] Dennis R. Barium meal techniques in dogs and cats. Pract. 1992;14:237–48. 10.1136/inpract.14.5.237.

[26] Hamid M, Ullah W, Ur Rashid M, Amjad W, Mukhtar M, Hurairah A. An esophagogram or tracheobronchogram? A review of barium sulfate aspiration. J Investig Med High Impact Case Rep. 2018;6. 10.1177/2324709618802872. 10.1177/2324709618802872 PMC6172932 30302346

[27] Vangara A, Gullapalli D, Do TV, Chan R, Kalafatis K, Ganti S. Unexpected barium aspiration. J Investig Med High Impact Case Rep. 2023;11. 10.1177/23247096231181867. 10.1177/23247096231181867 PMC10288401 37341445

[28] Mathisen DJ, Grillo HC, Wain JC, Hilgenberg AD. Management of acquired nonmalignant tracheoesophageal fistula. Ann Thorac Surg. 1991;52:759–65. 10.1016/0003-4975(91)91207-C. 1929626 10.1016/0003-4975(91)91207-c

[29] Han Y, Liu K, Li X, Wang X, Zohu Y, Gu Z, et al. Repair of massive stent-induced tracheoesophageal fistula. J Thorac Cardiovasc Surg. 2009;137:813–7. 10.1016/j.jtcvs.2008.07.050. 19327501 10.1016/j.jtcvs.2008.07.050

[30] Ke M, Wu X, Zeng J. The treatment strategy for tracheoesophageal fistula. JTD. 2015;7. 10.3978/j.issn.2072-1439.2015.12.11 10.3978/j.issn.2072-1439.2015.12.11 PMC4700364 26807286

[31] Yasuda JL, Staffa SJ, Ngo PD, Clark SJ, Jennings RW, Manfredi MA. Comparison of detection methods for tracheoesophageal fistulae with a novel method: capnography with CO₂ insufflation. J Pediatr Gastroenterol Nutr. 2020;70:e88–93. 10.1097/MPG.0000000000002647. 31990867 10.1097/MPG.0000000000002647

[32] Zhou C, Hu Y, Xiao Y, Yin W. Current treatment of tracheoesophageal fistula. Ther Adv Resp Dis. 2017;11:173–80. 10.1177/1753465816687518. Available from doi. 10.1177/1753465816687518 PMC5933624 28391759

[33] Engum SA, Grosfeld JL, West KW, Rescorla FJ, Scherer LR, Smith SD. Analysis of morbidity and mortality in 227 cases of esophageal Atresia and/or tracheoesophageal fistula. Arch Surg. 1995;130:502–8. 10.1001/archsurg.1995.01430050052008. 7748088 10.1001/archsurg.1995.01430050052008

[34] Bibas BJ, Guerreiro Cardoso PF, Minamoto H, Eloy-Pereira LP, Tamagno MF, Terra RM, et al. Surgical management of benign acquired tracheoesophageal fistulas: A ten-year experience. Ann Thorac Surg. 2016;102(4):1081–7. 10.1016/j.athoracsur.2016.04.029. 27329192 10.1016/j.athoracsur.2016.04.029

[35] Ramai D, Bivona A, Latson W, Ofosu A, Ofori E, Reddy M, et al. Endoscopic management of tracheoesophageal fistulas. Ann Gastroenterol. 2019;32:24–9. 10.20524/aog.2018.0321. 30598588 10.20524/aog.2018.0321 PMC6302189

[36] Balekuduru A, Dhande S, Venkateshappa L, Subbaraj S. Tracheoesophageal fistula: bridging the gap by over-the‐scope clip services. J Dig Endosc. 2019;9:188–92. 10.4103/jde.JDE_18_18.

[37] Jiang P, Liu J, Yu D, Jie B, Jiang S. Closure of nonmalignant tracheoesophageal fistula using an atrial septal defect occluder: case report and review of the literature. CVIR. 2015;38:1635–9. 10.1007/s00270-015-1147-7. 26048016 10.1007/s00270-015-1147-7

[38] Clements DN, McGill S, Beths T, Sullivan M. Tracheal perforation secondary to suture irritation in a dog following a ventral slot procedure. JSAP. 2003;44:313–5. 10.1111/j.1748-5827.2003.tb00160.x. 10.1111/j.1748-5827.2003.tb00160.x 12866929

[39] Walters KL, Knight RC. Diagnosis of a tracheal tear by use of an oxygen analyzer in a dog with cervical trauma. J Am Vet Med Assoc. 2021;259:880–4. 10.2460/javma.259.8.880. 34609190 10.2460/javma.259.8.880

